# Mitochondrial Genomes of *Streptopelia decaocto*: Insights into Columbidae Phylogeny

**DOI:** 10.3390/ani14152220

**Published:** 2024-07-31

**Authors:** Jiangyong Qu, Xiaofei Lu, Xindong Teng, Zhikai Xing, Shuang Wang, Chunyu Feng, Xumin Wang, Lijun Wang

**Affiliations:** 1 College of Life Science, Yantai University, Yantai 264005, China; qjy@ytu.edu.cn (J.Q.); luxiaofei18@outlook.com (X.L.); xingzhk@ytu.edu.cn (Z.X.); wangshuang0456@126.com (S.W.); 18869359884@163.com (C.F.); 2 Qingdao International Travel Healthcare Center, Qingdao 266071, China; tengxindeng@163.com

**Keywords:** mitogenome, phylogeny, *Streptopelia decaocto*, Columbidae

## Abstract

**Simple Summary:**

The present study aimed to characterize the complete mitochondrial genome of *Streptopelia decaocto* (Frivaldszky, 1838) and investigated the evolutionary relationships among species within the Columbidae family. The mtDNA full sequence length of *S. decaocto* was 17,160 bp. The mitogenome of *S. decaocto* comprised 13 PCGs, 22 tRNA genes, 2 rRNA genes, non-coding regions, and a control region. Analysis of the entire mtDNA of *S. decaocto* revealed consistently positive AT-skew values, except for nad3, nad6, and the D-loop. Conversely the GC-skew values were consistently negative, except for nad6. These findings suggest that *S. decaocto* belongs to the Columbinae subfamily.

**Abstract:**

In this research, the mitochondrial genome of the *Streptopelia decaocto* was sequenced and examined for the first time to enhance the comprehension of the phylogenetic relationships within the Columbidae. The complete mitochondrial genome of *Streptopelia decaocto* (17,160 bp) was structurally similar to the recognized members of the Columbidae family, but with minor differences in gene size and arrangement. The structural AT content was 54.12%. Additionally, 150 mitochondrial datasets, representing valid species, were amassed in this investigation. Maximum likelihood (ML) and Bayesian inference (BI) phylogenetic trees and evolutionary time relationships of species were reconstructed based on cytb gene sequences. The findings from the phylogenetic evaluations suggest that the *S. decaocto* was classified under the Columbinae subfamily, diverging from the Miocene approximately 8.1 million years ago, indicating intricate evolutionary connections with its close relatives, implying a history of species divergence and geographic isolation. The diversification of the Columbidae commenced during the Late Oligocene and extended into the Miocene. This exploration offers crucial molecular data for the *S. decaocto*, facilitating the systematic taxonomic examination of the Columbidae and Columbiformes, and establishing a scientific foundation for species preservation and genetic resource management.

## 1. Introduction

Columbiformes are among the oldest and most diverse birds in existence [1,2,3]. They are globally distributed, mainly in tropical and temperate regions, with approximately 42 genera and 305 species worldwide [4]. The Columbidae family is subdivided into five subfamilies, including Columbinae, Didunculinae, Gourinae, Otidiphabinae, and Treroninae [1]. Currently, most pigeon species are facing threats such as habitat loss, overhunting, species competition, diseases, and climate change. For example, populations of *Ptilinopus arcanus* are declining due to hunting and habitat destruction, and are classified as critically endangered [5]. *Ectopistes migratorius*, once distributed throughout North America, are now extinct due to habitat clearing and hunting [6]. The endangered status of these species highlights the biodiversity crisis caused by human activities. *Alectroenas pulcherrimus*, with a large population, is not yet vulnerable, but studies show a decline [7]. Birds like *Streptopelia decaocto*, which are extremely widespread with a remarkable diversity of habitats and are able to coexist with humans, are rare [7,8,9,10]. Their adaptive mechanisms remain unknown [11,12]. Understanding the evolutionary relationships of habituated species is crucial for conservation efforts, revealing their role in ecosystems and vulnerability to environmental change.

Molecular markers and DNA sequencing serve as effective tools for classifying taxonomy and phylogenetic relationships among species [2]. Within the mitochondrial genome, numerous genes could be utilized as molecular markers for phylogenetic analysis in birds, offering a wealth of genetic information [13]. The genes cytb, COI, and ND2, with a moderate evolutionary rate, are particularly suitable for studying intermediate order elements. Among these, the cytb gene stands out for its conservation and high homology [14]. The relatively rapid evolution of mitochondrial sequences could result in mutation accumulation along short internodes in phylogeny, limiting the information from nuclear markers [15]. Several authors have successfully constructed well-resolved phylogenies based on avian mitogenomes [16,17,18]. Currently, the Columbidae family comprises over 300 species, with only partial fragments of mitochondrial DNA (mtDNA) identified, and complete mitogenomes known for 34 species [1,19,20,21,22,23,24]. To address this gap, further identification of mitogenomes within the Columbidae family is essential to enhance the understanding of *S. decaocto* genome diversity, evolution, and genetic foundation of Columbidae [25]. These data will significantly contribute to further research on the Columbidae family.

This work aims to analyze the genetic structure of mitochondria and the taxonomic status of *S. decaocto* and to elucidate the phylogenetic relationships among Columbidae from a genomic perspective. The questions revolve around the evolution of mt genomes, their characteristics, and their potential impact on the evolution of Columbidae. By integrating the phylogenetic results with the timing of evolutionary changes, genetic components (such as introns, gene clusters, intergenic regions, and GC islands), and evolutionary events using dating, a comprehensive understanding was achieved.

The Columbidae family has a wide distribution and is abundant in China, making it a valuable subject for studying bird geographical distribution patterns and population ecological genetics. Genetic analyses of this bird family remain limited, with most studies focusing on endangered subspecies of other bird species within the family. This study utilized the mtDNA cytb gene as a molecular marker to explore the genetic diversity and structure of 148 species of the Columbidae family [26,27,28]. We also examined the relationship between genetic diversity across species, which has significant implications for understanding genetic diversity and population connectivity in bird populations amidst China’s urbanization and the impact of climate on bird migration.

## 2. Materials and Methods

### 2.1. Samples and Laboratory Analyses

For this research, single specimens of *S. decaocto* blood samples were obtained from a single sampling event conducted in Qinhuangdao in Hebei, China (39.95° N, 119.57° E). Blood was extracted from birds collected in the field (after collecting blood, the birds were still alive). Whole genomic DNA was extracted using the Qiagen DNeasy Blood and Tissues Kit (Valencia, CA, USA), following the manufacturer’s protocols.

### 2.2. Genome Sequencing and Annotation

DNA was extracted and sequenced from blood samples of live individuals. The freshly frozen blood samples stored in liquid nitrogen were used for DNA extraction [29]. Following DNA isolation, 1 µg of purified DNA fragment per sample was used to create short-insert libraries following Illumina’s instructions. The short fragments were sequenced using an Illumina Hiseq 4000 sequencing system at Total Genomics Solution (TGS) Institute in Shenzhen, China [30].

Before assembly, raw reads were first filtered to eliminate reads with adapters, those with a quality score below 20, those containing a percentage of uncalled based equal to or greater than 10%, and duplicated sequences. In *S. decaocto*, the mitogenome was reconstructed through a combination of de novo and reference-guided assemblies, involving three assembly steps [31]. Initially, the filtered reads were assembled into contigs using SOAP de novo 2.04 software [32]. Subsequently, the contigs were aligned to the reference genome (NC_031447.1) of *S. decaocto* using BLAST (≥80% similarity and query coverage). Lastly, the raw reads were mapped again to the assembled draft mitogenome, visualized using Organellar Genome DRAW v1.2 software [33], and local assembly helped fill most of the gaps.

The mitochondrial genes were annotated using an online DOGMA tool [34] with default parameters. This included identifying PCGs, tRNA genes, and rRNA genes. The base composition and skewness of the genome of *S. decaocto* were analyzed. AT content values were computed using DNAsp. The GC and AT skews describing codon bias were calculated as follows: AT skew = (A − T)/(A + T); GC skew = (G − C)/(G + C). The relative synonymous codon usage (RSCU) of each PCG was calculated using MEGA v6 [35]. The entire mitogenome sequence was examined for potentially tandem repeats using Tandem Repeats Finder. Circular maps of mitogenomes in *S. decaocto* were generated using OGD raw v1.2 [33]. This mitogenome has been deposited into the GenBank database under the accession number NC_037513 for *S. decaocto*. The web server MITOS (http://mitos.bioinf.uni-leipzig.de/index.py) (accessed on 25 July 2024) was used to predict the tRNA genes in the mitochondrial genome, applying the invertebrate genetic code.

### 2.3. Phylogenetic Analysis of the Cytb Gene

All currently available mitogenomes in Columbidae, except mitogenome sequence in *S. decaocto,* were used in phylogenetic analysis. Phylogenetic relationships were built based on nucleotide sequences of 13 PCGs. Sequences were aligned using MAFFT (Ver. 7.0) and mtPCGs were spliced to form a matrix of tandem sequences, and the optimal partitioning model was determined using ModelFinder (Ver. 2.2.0) based on the Bayesian information criterion (BIC). The 13 mtPCGs were aligned to form a 17,160 bp long sequence, and the optimal partitions were calculated by ModelFinder: ① atp6, nad1, nad2, nad3, nad4, and nad5; ② atp8; ③ cox1, cox2, cox3, and nad4L; ④ cytb, nad1; ⑤ nad6. The optimal model for all 5 partitions was GTR + F + I + G. Final nucleotide sequences of each gene were linked into single contigs for phylogenetic analyses. The best-fit models of nucleotide substitution were identified using IQ-TREE Model Selection with the +R option (FreeRate model) that generalizes the +G model by relaxing the assumption of gamma-distributed rates [36]. Maximum likelihood (ML) was selected to construct the phylogenetic tree; following that, ML bootstrapping analysis was performed using RAxML v8.2.12 to reconstruct a phylogenetic tree [37], with 1000 bootstraps for node reliability estimation. A phylogenetic tree was constructed based on the mitogenome of *S. decaocto* and thirty-five available Columbidae species (Table S4), showing the relative position of *S. decaocto* within the Columbidae clade.

To further analyze the phylogenetic relationships of *S. decaocto* in Columbidae, the phylogenetic tree was constructed based on all available cytb genes from the Columbidae family. Two species from one species of Phasianidae and one species of Pteroclidae were used as outgroups. ML and BI methods were used for phylogenetic analyses. cytb gene sequences were aligned using Clustal X with default parameters. The ML tree was generated using PhyML [37]. Best-fit nucleotide substitution models for partition were selected using jModelTest (GTR + I + G) [36], and the bootstrap probability value for ML trees was calculated with 1000 bootstrap replicates. A BI tree was generated using MrBayes v3.2.5 [38] with the best-fit selected by MrModelTest [39], running four simultaneous Markov Monte Carlo chains for 50,000,000 generations, sampling a tree every 100 generations. Resulting phylogenetic trees were visualized using TreeView.

The phylogenetic relationships were estimated based on all available cytb genes from the Columbidae family using BEAST 1.8.1 [40]. A normal uncorrelated relaxed clock was assumed for each partition in BEAST, and a birth–death speciation process was assumed for the tree prior. To calibrate the molecular clock, a normal prior was placed on the age of the divergence between Columbiformes and Pterocliformes of 55 Mya ± 15 Mya [41]. A fixed mean substitution rate of 1.6% change per million years (Myr-1) was used, obtained by averaging the cytb divergence rates and dividing by two. Analyses ran for 30 million states, sampling trees and model parameters every 3000 states. The first 30% was discarded as burn-in. To ensure result consistency, each run was repeated at least three times with different random number seeds. Effective sample size values for parameters of interest were all over 300. Results were visualized and checked using Tracer v 1.6.

## 3. Results

### 3.1. Genome or Ganization: Structure and Composition

**Figure 1:**
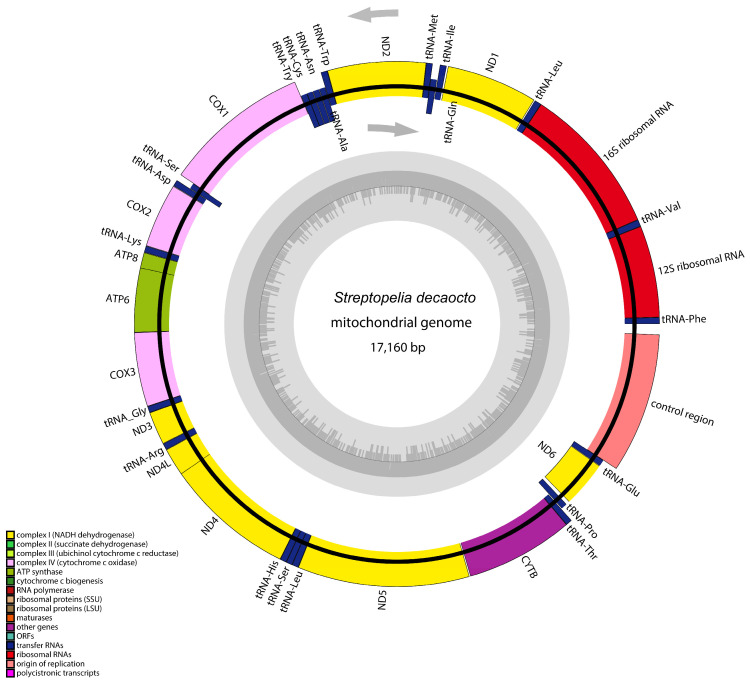
Organization of the mitochondrial genome of *S. decaocto*. Genes for proteins and rRNA (rrnS and rrnL) are listed in abbreviations. Transfer RNAs are represented by one letter amino acid code.

There was a mitochondrial genome with a length of 17,160 bp. Its structural organization is illustrated in Figure 1 and Table S1. The mitogenome of *S. decaocto* consisted of 13 PCGs, 22 tRNA genes, 2 rRNA genes, non-coding regions, and a control region. In this specie, nine genes and twenty-eight genes were transcribed from the L-strand and the H-strand of the molecules, respectively. The *S. decaocto* mitogenome comprised 22 non-coding regions of 1–194 bp, totaling 771 bp. There were thirteen occurrences of overlaps with a combined length of 83 bp in this species. Additionally, in *S. decaocto*, the longest non-coding region was located between D-loop and trnF, followed by the one located between trnL1tag and nad5. The longest overlapping region was found between nad4 and trnH.

The mitogenome analyzed in this study exhibited a slight bias in nucleotide composition. The overall mtDNA base composition of *S. decaocto* was A 30.09%, T 24.03%, G 13.78%, and C 32.10% with an AT content of 54.12%. Skewness was further evaluated in different gene regions in *S. decaocto* to assess the base bias of these mitogenomes. The results indicated that AT-skew values were all positive in *S. decaocto*, except for nad3, nad6, and D-loop, while values of the GC-skew were all negative, except for nad6 (Table S1).

### 3.2. The Protein-Coding Genes

**Figure 2:**
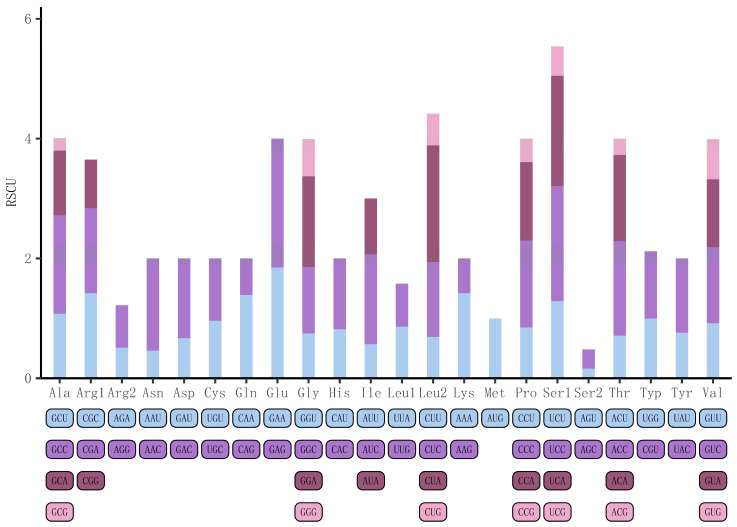
Relative synonymous codon usage (RSCU) in the mitogenomes of *S. decaocto*.

The total length of the PCGs in *S. decaocto* was 11,043 bp, accounting for 64.35%. The overall AT content of the 13 PCGs in *S. decaocto* was 53.79%, ranging from 50.96% (nad6) to 56.96% (nad2). All 13 PCGs in *S. decaocto* initiated with the common start codon ATG, except for coxI, nad3, and nad5, which started with ATA. The PCGs used five types of stop codons, including AGG for coxI; TAA for coxII, atp6, atp8, nad4l, and cytb; TCA for coxIII; AGA for nad1, nad3, nad4, and nad5; and TAG for nad2 and nad6. A total of 3680 amino acids were encoded in the *S. decaocto* mitogenome. Amino acids encoded by AT-rich codon families were more frequent than those encoded by GC-rich codon families, similar to other known Columbidae species (Figure 2). The most frequently occurring codon was UCC, followed by UCA, CCC, and CCA (Table S3).

### 3.3. Transfer RNA and Ribosomal RNA Genes

**Figure 3:**
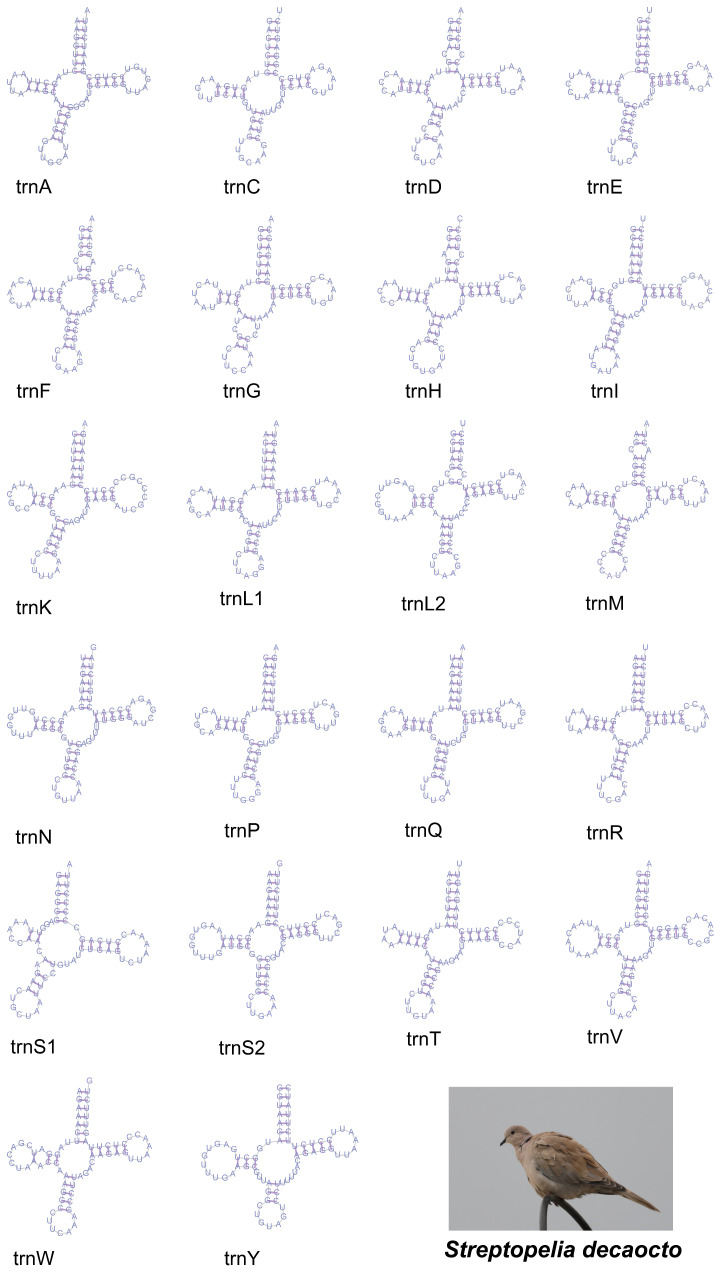
Secondary structure of the tRNA genes in the mitogenome of *S. decaocto*.

The set of 22 tRNA genes included 2 tRNAs for Ser and Leu each, and 1 tRNA for each of the other 18 amino acids (Figure 3), of which 8 were located on the L-strand and others on the H-strand (Table S2). The *S. decaocto* mitogenome had 1547 bp of tRNA genes, with an AT content of 52.00%, AT skew of 0.22, and GC skew of −0.19. And all tRNAs could fold into a complete coverleaf, expect for Trna-Ser (AGY) due to lacking the DHU stem. Variations in nucleotide sequences from homologs were mainly found at non-pair sites within the DHU and TΨC loops [42,43].

### 3.4. Anticodon, Non-Coding Regions, and Control Regions

A total of 22 non-coding regions, totaling 771 bp, were interspersed throughout the *S. decaocto* mitogenome. The AT content of non-coding regions in *S. decaocto* was 54.56%, with a positive AT-skew (0.40) and a negative GC-skew (−0.91) (Table S2). The control region was flanked by trnE and trnF, positioned similarly to other avians. The control region’s length of *S. decaocto* was 1317, with an AT content of control regions of 57.40%, negative AT-skew, and GC-skew (Table S2). Similar to other birds, the control region had three structural domains: the highly variable peripheral structural domains I and III (ranging from 100 to 300 base pairs) and the centrally conserved structural domain II (approximately 200 to 300 base pairs).

### 3.5. Mitogenomes of Columbidae Species

Mitochondrial non-coding regions covered 10.89% of the *S. decaocto* genome. Overlapping genes and tandem repeat sequences were identified in the *S. decaocto* mitogenome, with 13 occurrences of 1 to 47 bp overlaps totaling 83 bp. The largest repeating sequence of 47 bp was located between nad4 and trnH. Additionally, there was one positive-strand tandem repeat in *S. decaocto* with the sequence consensus (CAAACAAACAGA).

### 3.6. Phylogenetic Analyses

**Figure 4:**
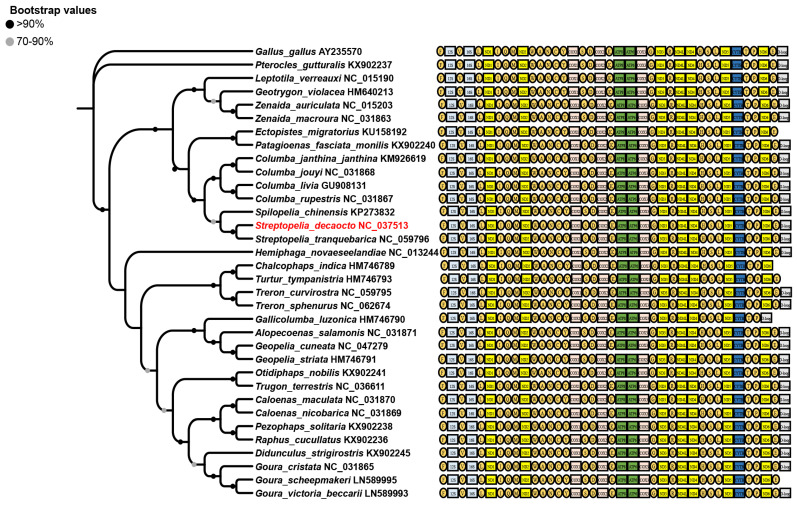
Linear representation of the mitochondrial gene arrangement in thirty-five Columbidae species, including *S. decaocto* (in red), plus two outgroup species as a reference. Genes for proteins and rRNA (rrnS and rrnL) are listed in abbreviations. Transfer RNAs are represented by one letter code of amino acids. Non-coding regions are not displayed, and gene segments are not drawn to scale. Phylogenetic tree is generated based on the sequences of 13 PCGs of the mitogenome using ML analysis. The nodes represent the ML bootstrap proportions, with 1000 bootstrap replicates.

**Figure 5:**
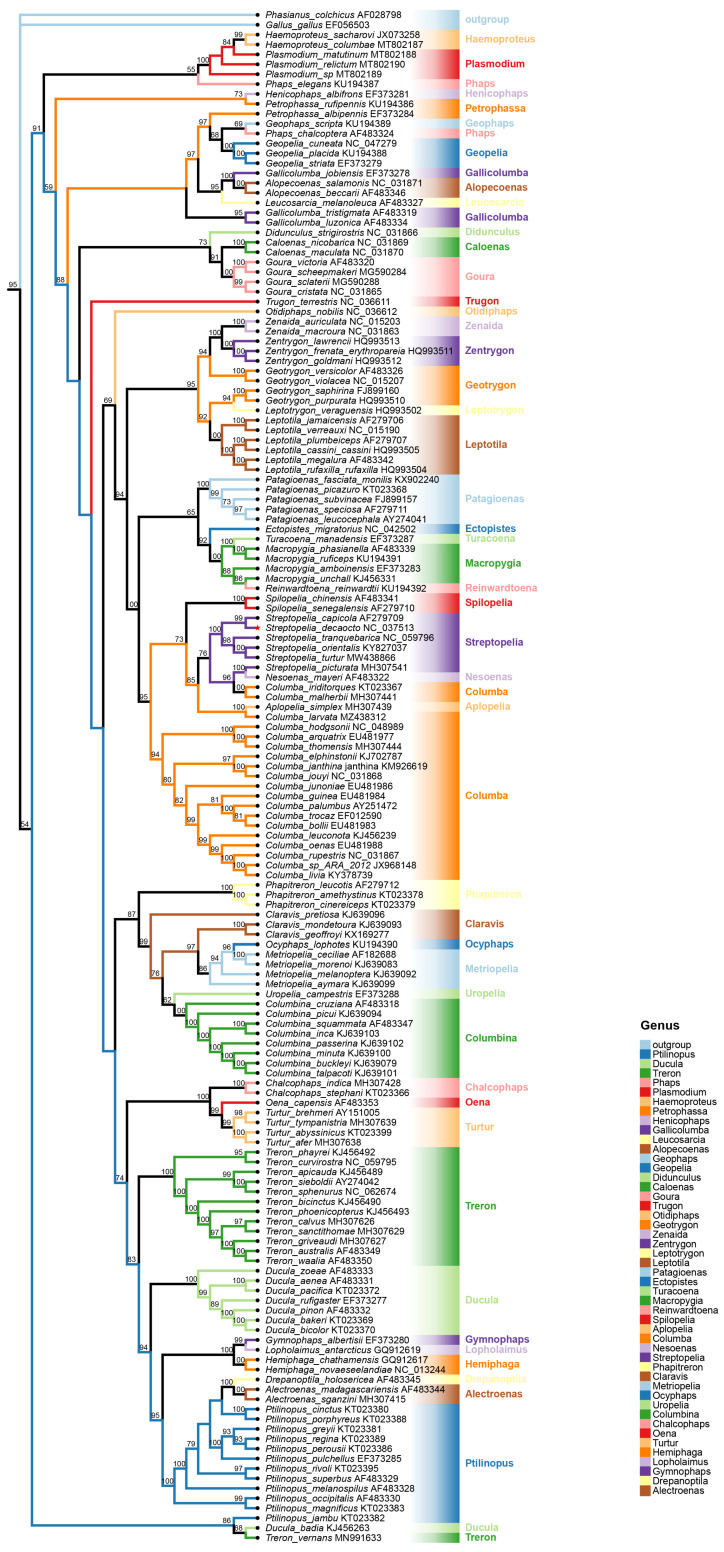
Phylogenetic relationships in Columbidae based on cytb sequences. Phylogenetic tree constructed by BI and ML methods, based on all available Columbidae cytb genes, including *S. decaocto* (in red). Species names are followed by the GenBank accession number, statistical support is shown next to branches, and the tree scale is included in the figure. Taxonomic identity is shown to the right: genus.

We utilized ML and Bayesian inference (BI) methods, and both ML and BI tree inference yielded identical tree topologies (Figure 4). Columbidae was found to be divided into four subfamilies, with the largest number of species belonging to the Columbinae subfamily, followed by the Trerononae subfamily. The analyses revealed that *S. decaocto* belongs to the Columbinae subfamily. It was evident that *S. orientalis* and *S. turtur* shared a close ancestor with *S. decaocto* and *S. capicola*, forming a monophyletic clade supported by both ML and BI analyses (bootstrap = 81; posterior probability = 1) (Figure 5). Additionally, species in the *Columba* genus formed a clade with strong posterior probability and bootstrap support, and together these species formed another well-supported clade with species in the *Streptopelia* genus.

**Figure 6:**
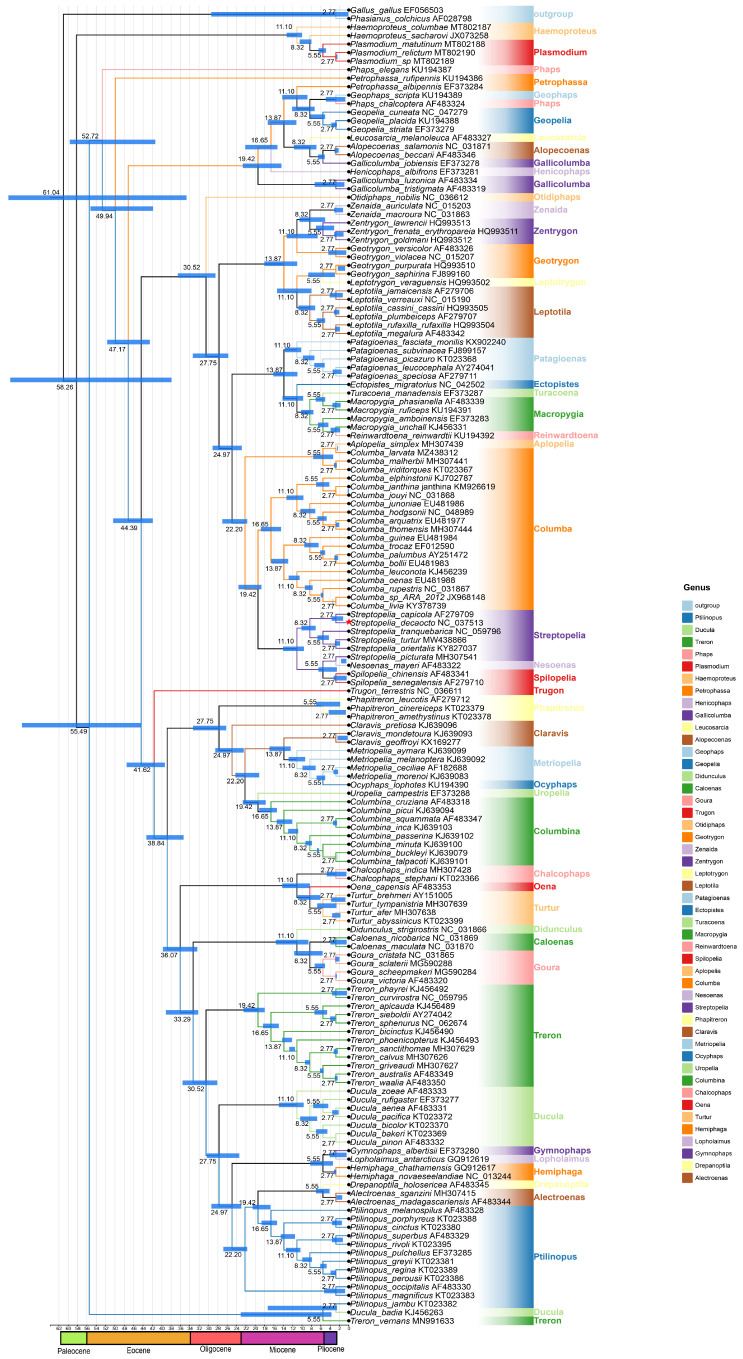
Divergence time estimation for Columbidae inferred with BEAST based on *Cytb*. The 95% highest posterior distribution (HPD) is reported using blue bars. *S. decaocto* (in red).

The combination of recent genome-scale analyses of avian evolution and our new data set of cytb genes provides an opportunity to recalibrate the timing of the origin of and radiation within the Columbidae, including several extinct or extremely rare species. This study found that doves most likely began to diversify during the late Oligocene, and continued to diversify into the Miocene (Figure 6), showing more recent diversification than previously suggested. The transition from the Eocene to the Oligocene coincided with a period of substantial global cooling and associated geological changes, potentially explaining the rapid diversification within Columbidae, and estimate that the Columbidae and outgroup species (Table S5) diverged around 58.5 Mya, during the Paleocene/Eocene transition (Figure 6). This study found that the *S. decaocto* and *S. capicola* diverged around 6.87 Mya.

## 4. Discussion

The mitogenome structure of *S. decaocto* was similar to those of other doves, but an extra cytosine was inserted at position 174 in nad3 in this species [28]. The relative synonymous codon usage (RSCU) in the *S. decaocto* mitogenome was similar to that in other Columbidae species (Figure 2). The codon usage bias of mitogenomes was also observed in vertebrates, where the two strands were exposed to different mutational pressures during replication, leading to an increased frequency of A and C in the (+) strand (or L-strand in the case of vertebrates) [44]. This phenomenon was also observed in this species in the current study. As is typical of most metazoans, the mitogenome of *S. decaocto* contains 22 tRNAs and 2 rRNAs (12S rRNA and 16S rRNA). In vertebrate mtDNAs, the most frequently used codon in a degenerate codon family precisely matches the corresponding tRNA anticodon, a phenomenon known as codon–anticodon adaptation. The mitogenomes in *S. decaocto* shared the same tRNA anticodons as vertebrates (Figure 3), suggesting that the evolution of anticodon in the mitogenome may be influenced by similar mechanistic forces. In addition, gene spacers and gene overlap were present in the mitochondrial genome of *S. decaocto*, and these features were highly similar to those of other birds in the Columbidae family [22,45]. Gene overlap was prevalent in vertebrate mitochondrial genomes, and the occurrence of this phenomenon allowed efficient base reuse, reduced DNA replication time, and also served to regulate selection [46].

The sizes of mitogenomes of Columbidae species were not remarkable compared with known mitogenomes, and there were no significant differences observed between species. While Columbidae mitogenomes were on average 16.8 kb in size, ranging from 15.2 kb (*Gallicolumba luzonica*) to 17.5 kb *(Columba janthina*) among the thirty-five genomes sequenced to date (https://www.ncbi.nlm.nih.gov/) (accessed on 25 July 2024), the *S. decaocto* mitogenome was 17,160 bp in length. The phylogenetic tree (Figure 4) was constructed based on the mitogenomes of *S. decaocto* and the thirty-five available Columbidae species Although it was challenging to reconstruct with the limited genomes, the diversity in genome size among different species reflected a dynamic history of species expansion. The genome size was significantly correlated with the combined length of coxI-III, the length of cytb, and the combined length of rRNAs (rrnS and rrnL). And these six genes were absolutely necessary for oxidative phosphorylation [47]. And there were two hypotheses about relationships between gene length and genome size. The first hypothesis was proposed by Müller’s ratchet, which states that redox reactions produce oxygen free radicals during respiration in many mitochondria, leading to a higher mutation rate than their corresponding nuclear DNA. These deleterious mutations could accumulate and cause a mutational meltdown if recombination never occurs. Another hypothesis was the replication advantage hypothesis. It was suggested that a smaller mitogenome would be selected in competition within cells due to its faster duplication rate. This generally contributes to the elimination of redundant cytoplasmic genes through selective deletions in organelles [48]. Both hypotheses predict a relationship between genome size and gene length. The observed variations in evolutionary relationships between morphology-based, gene-based and mitogenome-based may be due to the incorrect classification of the conventional morphology-based. Furthermore, as more mitogenomes of Columbidae species become available, the phylogenetic relationships among the Columbidae will become clearer.

In this study, a phylogenetic tree of birds in the family Columbidae was constructed using cytb gene sequences. The Columbidae phylogenetic tree was established through maximum likelihood (ML) and Bayesian (BI) methods using 13 PCGs genes (Figure 5) [49]. It was determined that the oriental chained spider is in the same taxon as the ten-tailed chained spider and the middle-tailed chained spider, which aligns with this study’s findings. The Columbidae phylogenetic tree was constructed utilizing the neighbor-joining (NJ) method with COI gene sequences [50]. Results indicated that *Streptopelia orientalis* grouped with *Streptopelia chinensis*, *Streptopelia tranquebarica*, and *Streptopelia decaocto*, consistent with this study. *Streptopelia* and *Columba* species formed sister groups with significant node support values, in line with past research [49]. Furthermore, the phylogenetic relationships of the 12 PCGs based on BI analysis [28] showed that *Spilopelia chinensis* and *Columba livia* were closely related sister branches (a posteriori probability value of 1).

This study utilized timescales estimated by Jarvis et al. [41] and Prum et al. [51], which were consistent with each other regarding the diversification timing of Columbidae. Recalibration of the origin and radiation timing was performed based on all available cytb genes in the Columbidae family, with one Phasianidae and one Pteroclidae species as outgroups, using BEAST 1.8.1 [40]. The common ancestor of Columbidae diverged in the early Eocene around 55.0 Mya (Figure 6). In Columbidae, all species diverged from the Oligocene, except *Ducula badia* and *Ptilinopus jambu*, which diverged from the Eocene around 41.7 Mya. Species in the *Streptopelia* genus specifically diverged in the middle Miocene, sharing a common ancestor with *Columba* and diverging around 14.6 Mya. Rapid diversification may lead to changes in gene trees due to the effects of incomplete lineage sorting [1], which could result in the inference of different phylogenies for different loci. In the future, utilizing additional calibration points will enhance the accuracy of node age estimates, indicating the direction for future research.

## 5. Conclusions

The characterizations of the evolution and structural organization of the *S. decaocto* mitogenome were analyzed and compared with other Columbidae mitogenomes. This study discussed results with a particular emphasis on the genome size variation and structural organization in the Columbidae family. Meanwhile, we reconstructed the phylogenetic relationships of thirty-five Columbidae species based on thirteen PCGs, enabling a better understanding of the ancestral organization of Columbidae mitogenomes.

## Data Availability

The genome sequence data that support the findings of this study are openly available in GenBank of NCBI at https://www.ncbi.nlm.nih.gov/ under the accession no. NC_037513 (or KY827036). The associated Bioproject, SRA, and Bio-Sample number are PRJNA927338, SAMN39090883, and SUB14110933, respectively.

## Supplementary Materials

The following supporting information can be downloaded at: https://www.mdpi.com/article/10.3390/ani14152220/s1, Table S1: AT-content, AT-skew and GC-skew for mitochondrial genes in *Streptopelia decaocto*; Table S2: Gene features and organization of *Streptopelia decaocto*; Table S3: Codon usage of *Streptopelia decaocto* PCGs; Table S4: Complete mitochondrial genomes and their GenBank accession number used for phylogenetic analysis; Table S5: Taxonomic classification of the species chosen for all available Cytb genes in Columbidae family and one Phasianidae species and one Pteroclidae specie as outgroup in Columbiformes.
