# Supplementary material for: Mitochondrial Genomes of Streptopelia decaocto: Insights into Columbidae Phylogeny
Source: Animals (Basel). 2024 Jul 31;14(15):2220. doi: 10.3390/ani14152220 (PMC11310995; doi:10.3390/ani14152220)
Supplement: Supplementary file 1 [file animals-14-02220-s001.zip › animals-3072801-supplementary.pdf]

Supporting Information

Mitochondrial Genomes of *Streptopelia decaocto*: Insights into Columbidae Phylogeny

Jiangyong Qu [1,†], Xiaofei Lu [1,†], Xindong Teng [2], Zhikai Xing [1], Shuang Wang [1], Chunyu Feng [1], Xumin Wang [1,*] and Lijun Wang [1,*]

[1] College of Life Science, Yantai University, Yantai 264005, China; qjy@ytu.edu.cn (J.Q.); luxiaofei18@outlook.com (X.L.); xingzhk@ytu.edu.cn (Z.X.); wangshuang0456@126.com (S.W.); 18869359884@163.com (C.F.)
[2] Qingdao International Travel Healthcare Center, Qingdao 266071, China; tengxindeng@163.com
* Correspondence: wangxm@ytu.edu.cn (X.W.); wanglijun@ytu.edu.cn (L.W.)
† The two authors contributed equally to this paper.

This file contains supplementary 5 tables:

Table S1 AT-content, AT-skew and GC-skew for mitochondrial genes in *Streptopelia decaocto*.

Table S2. Gene features and organization of *Streptopelia decaocto*.

Table S3. Codon usage of Streptopelia decaocto PCGs.

Table S4. Complete mitochondrial genomes and their GenBank accession number used for phylogenetic analysis

Table S5. Taxonomic classification of the species chosen for all available Cytb genes in Columbidae family and one Phasianidae species and one Pteroclidae specie as outgroup in Columbiformes.

Table S1. AT-content, AT-skew and GC-skew for mitochondrial genes in *Streptopelia decaocto*.

Feature	(A+T) %	ATskew	GC skew
Whole genome	54.12%	0. 11	-0.4
Protein-coding genes	53.79%	0.04	-0.41
cox1	52.35%	0.02	-0.35
cox2	53.65%	0. 12	-0.38
cox3	53.26%	0.04	-0.36
cytb	53. 19%	0.01	-0.45
nad1	53.00%	0	-0.45
nad2	56.96%	0. 15	-0.54
nad3	54.73%	-0.09	-0.56
nad4	54.53%	0. 11	-0.52
nad4l	55.22%	0.01	-0.43
nad5	53.91%	0. 14	-0.5
nad6	50.96%	-0.6	0.61
atp6	53.65%	0.08	-0.56
atp8	56.55%	0. 12	-0.7
tRNAs	52.00%	0.22	-0.19
rrnS	54.90%	0.23	-0.19
rrnL	56.43%	0.04	-0.01
D-loop	57.40%	-0.07	-0.33
NCR	54.56%	0.4	-0.91

Table S2. Gene features and organization of *Streptopelia decaocto*.

Element	Strand	Position	Size (bp)		Intergenic	Start	Stop
			nt	aa	nucleotide	codon	codon
cox*1*	H	1-1,467	1,467	488	85	ATA	AGG
t*rnS2TGA*	L	1,459-1,532	74		-9		
t*rnD*	H	1,535-1,603	69		2		
cox*2*	H	1,606-2,289	684	227	2		
t*rnK*	H	2,291-2,361	71		1		
atp*8*	H	2,363-2,530	168	55	1	ATG	TAA
atp*6*	H	2,521-3,204	684	227	-10	ATG	TAA
cox*3*	H	3,204-3,986	783	261	-1	ATG	TCA
t*rnG*	H	3,988-4,056	69		1		
nad*3*	H	4,063-4,263	201	66	6	ATA	AGA
t*rnR*	H	4,410-4,478	69		146		
nad*4l*	H	4,480-4,776	297	98	1	ATG	TAA
nad*4*	H	4,770-6,194	1,425	474	-7	ATG	AGA
trn*H*	H	6,148-6,216	69		-47		
t*rnS1GCT*	H	6,217-6,282	66		0		
t*rnL1TAG*	H	6,282-6,352	71		-1		
nad*5*	H	6,509-8,170	1,662	553	156	ATA	AGA
Cy*tb*	H	8,181-9,323	1,143	380	10	ATG	TAA
t*rnT*	H	9,323-9,391	69		-1		
t*rnP*	L	9,399-9,468	70		7		
nad*6*	L	9,507-10,028	522	173	38	ATG	TAG
t*rnE*	L	10,032-10,102	71		3		
D-*loop*		10,186-11,502	1,317		83		
t*rnF*	H	11,697-11,766	70		194		
r*rnS*	H	11,766-12,738	973		-1		
t*rnV*	H	12,738-12,810	73		-1		
r*rnL*	H	12,810-14,401	1,592		-1		
t*rnL2TAA*	H	14,401-14,474	74		-1		
nad*1*	H	14,485-15,450	966	321	10	ATG	AGA
t*rnI*	H	15,466-15,536	71		15		
trn*Q*	L	15,542-15,612	71		5		
t*rnM*	H	15,612-15,680	69		-1		
nad*2*	H	15,681-16,721	1,041	346	0	ATG	TAG
t*rnW*	H	16,720-16,790	71		-2		
t*rnA*	L	16,792-16,860	69		1		
t*rnN*	L	16,863-16,935	73		2		
t*rnC*	L	16,938-17,004	67		2		
t*rnY*	L	17,005-17,075	71		0		

Table S3. Codon usage of Streptopelia decaocto PCGs.

Codon	N	RSCU
UAU(Y)	67	0.76
UAC(Y)	110	1.24
UAA(*)	116	1.29
UAG(*)	101	1. 13
CAU(H)	61	0.82
CAC(H)	88	1.18
CAA(Q)	124	1.39
CAG(Q)	54	0.61
AAU(N)	22	0.46
AAC(N)	74	1.54
AAA(K)	75	1.42
AAG(K)	31	0.58
GAU(D)	28	0.67
GAC(D)	56	1.33
GAA(E)	66	1.25
GAG(E)	40	0.75
UGU(C)	23	0.96
UGC(C)	25	1.04
UGA(*)	52	0.58
UGG(W)	36	1
CGU(R)	11	1.12
CGC(R)	14	1.42
CGA(R)	14	1.42
CGG(R)	8	0.81
AGU(S)	12	0.16
AGC(S)	24	0.32
AGA(R)	5	0.51

AGG(R)	7	0.71
GGU(G)	23	0.75
GGC(G)	34	1. 11
GGA(G)	46	1.51
GGG(G)	19	0.62

N: number of occurrence of the codon. RSCU: relative synonymous codon usage.

Table S4. Complete mitochondrial genomes and their GenBank accession number used for phylogenetic analysis

ID	NCBI accession	Total reads	Authors
Gallus_gallus	AY235570.1	16,784bp	Linnaeus, 1758 **(outgroup)**
Pterocles_gutturalis	KX902237.1	15,637bp	Smith, 1836
Leptotila_verreauxi	NC_015190.1	17,176bp	Bonaparte, 1855
Geotrygon_violacea	HM640213.1	16,864bp	Temminck, 1809
Zenaida_auriculata	NC_015203.1	16,781bp	des Murs, MAPO 1847
Zenaida_macroura	NC_031863.1	17,132bp	Linnaeus, C 1758
Ectopistes_migratorius	KU158192.1	17,014bp	Linnaeus, 1766
Patagioenas_fasciata_monilis	KX902240.1	16,967bp	Vigors, 1839
Columba_janthina_janthina	KM926619.1	17,469bp	Temminck, 1830
Columba_jouyi	NC_031868.1	17,179bp	Stejneger, 1887
Columba_livia	GU908131.1	17,229bp	Gmelin, 1789
Columba_rupestris	NC_031867.1	17,201bp	Pallas, 1811
Spilopelia_chinensis	KP273832.1	16,966bp	Scopoli, 1768
Streptopelia_decaocto	NC_037513.1	17,160bp	Frivaldszky, 1838
Streptopelia_orientalis	KY827037.1	17,102bp	Latham, 1790
Streptopelia_tranquebarica	NC_059796.1	17,431bp	Hermann, 1804
Hemiphaga_novaeseelandiae	NC_013244.1	17,264bp	Gmelin, JF, 1789
Chalcophaps_indica	HM746789.1	15,363bp	Linnaeus, 1758
Turtur_tympanistria	HM746793.1	15,557bp	Temminck, 1809
Treron_curvirostra	NC_059795.1	17,540bp	Gmelin, JF, 1789
Treron_sphenurus	NC_062674.1	18,919bp	Vigors, 1832
Gallicolumba_luzonica	HM746790.1	15,192bp	Scopoli, 1786
Alopecoenas_salamonis	NC_031871.1	17,141bp	Ramsay, EP, 1882
Geopelia_cuneata	NC_047279.1	17,880bp	Latham, 1801
Geopelia_striata	HM746791.1	15,859bp	Linnaeus, 1766
Otidiphaps_nobilis	KX902241.1	16,570bp	Gould, 1870
Trugon_terrestris	NC_036611.1	17,405bp	Gray, 1849
Caloenas_maculata	NC_031870.1	17,036bp	Gmelin, 1789
Caloenas_nicobarica	NC_031869.1	17,090bp	Linnaeus, 1758
Pezophaps_solitaria	KX902238.1	16,644bp	Gmelin, 1789
Raphus_cucullatus	KX902236.1	17,092bp	Linnaeus, 1758
Didunculus_strigirostris	KX902245.1	17,071bp	Jardine, 1845
Goura_cristata	NC_031865.1	17,082bp	Pallas, 1764
Goura_scheepmakeri	LN589995.1	17,131bp	Finsch, 1876
Goura_victoria_beccarii	LN589993.1	17,125bp	Salvadori, 1876

Table S5. Taxonomic classification of species selected from all species containing the Cytb gene: Columbidae, and as outgroups, a species of Phasianidae and a species of Pteroclidae in the Columbiformes.

Species	NCBI accession	Total reads	Authors
Phasianus_colchicus	AF028798.1	1,143 bp	Linnaeus, 1758**(outgroup)**
Gallus_gallus	EF056503.1	909 bp	Linnaeus, 1758 **(outgroup)**
Ptilinopus_jambu	KT023382.1	919 bp	Gmelin, 1789
Ducula_badia	KJ456263.1	1,143bp	Raffles, 1822
Treron_vernans	MN991633.1	785bp	Linnaeus, 1771
Phaps_elegans	KU194387.1	1,057bp	Temminck, 1809
Petrophassa_rufipennis	KU194386.1	1,055bp	Collett, 1898
Henicophaps_albifrons	EF373281.1	1,032bp	Gray, 1862
Gallicolumba_tristigmata	AF483319.1	1,052bp	Bonaparte, 1855
Gallicolumba_luzonica	AF483334.1	1,052bp	Scopoli, 1786
Leucosarcia_melanoleuca	AF483327.1	1,052bp	Latham, 1801
Gallicolumba_jobiensis	EF373278.1	1,044bp	Meyer, 1875
Alopecoenas_salamonis	NC_031871.1	1,7141bp	Ramsay, EP, 1882
Alopecoenas_beccarii	AF483346.1	1,052bp	Salvadori, 1876
Petrophassa_albipennis	EF373284.1	1,044bp	Gould, 1841
Geophaps_scripta	KU194389.1	546bp	Temminck, 1821
Phaps_chalcoptera	AF483324.1	1,052bp	Latham, 1790
Geopelia_cuneata	NC_047279.1	1,7880bp	Latham, 1801
Geopelia_placida	KU194388.1	927bp	Gould, 1844
Geopelia_striata	EF373279.1	1,042bp	Linnaeus, 1766
Didunculus_strigirostris	NC_031866.1	1,7071bp	Jardine, 1845
Caloenas_nicobarica	NC_031869.1	1,7090bp	Linnaeus, 1758
Caloenas_maculata	NC_031870.1	1,7036bp	Gmelin, 1789
Goura_victoria	AF483320.1	1,052bp	Fraser, 1844
Goura_scheepmakeri	MG590284.1	1,7224bp	Finsch, 1876
Goura_sclaterii	MG590288.1	1,8242bp	Salvadori, 1876
Goura_cristata	NC_031865.1	1,7082bp	Pallas, 1764
Trugon_terrestris	NC_036611.1	1,7405bp	Gray, 1849
Otidiphaps_nobilis	NC_036612.1	1,7346bp	Gould, 1870
Geotrygon_versicolor	AF483326.1	1,052bp	Lafresnaye, 1846
Geotrygon_violacea	NC_015207.1	1,6864bp	Temminck, 1809
Zenaida_auriculata	NC_015203.1	1,6781bp	Des Murs, 1847
Zenaida_macroura	NC_031863.1	1,7132bp	Linnaeus, C 1758
Zentrygon_lawrencii	HQ993513.1	1,045bp	Salvin, 1874
Zentrygon_frenata_erythropareia	HQ993511.1	1,045bp	Salvadori, 1893
Zentrygon_goldmani	HQ993512.1	1,037bp	Nelson, 1912
Leptotrygon_veraguensis	HQ993502.1	1,045bp	Lawrence, 1867
Geotrygon_saphirina	FJ899160.1	1,034bp	Bonaparte, 1855
Geotrygon_purpurata	HQ993510.1	1,045bp	Salvin, 1878

Leptotila_jamaicensis	AF279706.1	1,017bp	Linnaeus, 1766
Leptotila_verreauxi	NC_015190.1	17,176bp	Bonaparte, 1855
Leptotila_plumbeiceps	AF279707.1	1,045bp	Sclater & Salvin, 1868
Leptotila_cassini_cassini	HQ993505.1	1,045bp	Lawrence, 1867
Leptotila_megalura	AF483342.1	1,044bp	Sclater & Salvin, 1879
Leptotila_rufaxilla_rufaxilla	HQ993504.1	1,045bp	Richard, L-C; Bernard, J-P 1792
Patagioenas_fasciata_monilis	KX902240.1	16,967bp	Vigors, 1839
Patagioenas_picazuro	KT023368.1	354bp	Temminck, 1813
Patagioenas_subvinacea	FJ899157.1	1,035bp	Lawrence, 1868
Patagioenas_speciosa	AF279711.1	1,045bp	Gmelin, 1789
Patagioenas_leucocephala	AY274041.1	1,285bp	Linnaeus, 1758
Ectopistes_migratorius	NC_042502.1	17,026bp	Linnaeus, 1766
Turacoena_manadensis	EF373287.1	1,044bp	Quoy, JRC; Gaimard, JP 1832
Macropygia_phasianella	AF483339.1	1,044bp	Temminck, 1821
Macropygia_ruficeps	KU194391.1	914bp	Temminck, 1835
Macropygia_amboinensis	EF373283.1	1,035bp	Linnaeus, 1766
Macropygia_unchall	KJ456331.1	1,143bp	Wagler, 1827
Reinwardtoena_reinwardtii	KU194392.1	1,052bp	Temminck, CJ 1824
Spilopelia_chinensis	AF483341.1	1,044bp	Scopoli, 1768
Spilopelia_senegalensis	AF279710.1	1,045bp	Linnaeus, 1766
Columba_iriditorques	KT023367.1	1,013bp	Cassin, 1856
Columba_malherbii	MH307441.1	976bp	Verreaux & Verreaux, 1851
Nesoenas_mayeri	AF483322.1	1,052bp	Prevost, 1843
Streptopelia_picturata	MH307541.1	1,105bp	Temminck, 1813
Aplopelia_simplex	MH307439.1	944bp	Hartlaub, 1849
Columba_larvata	MZ438312.1	472bp	Temminck, 1809
Streptopelia_capicola	AF279709.1	1,045bp	Sundevall, CJ 1857
Streptopelia_decaocto	NC_037513.1	17,160bp	Frivaldszky, 1838
Streptopelia_tranquebarica	NC_059796.1	17,431bp	Hermann, 1804
Streptopelia_orientalis	KY827037.1	17,102bp	Latham, 1790
Streptopelia_turtur	MW438866.1	892bp	Linnaeus, 1758
Columba_hodgsonii	NC_048989.1	17,477bp	Vigors, 1832
Columba_arquatrix	EU481977.1	972bp	Temminck, 1809
Columba_thomensis	MH307444.1	1,105bp	Bocage, 1888
Columba_elphinstonii	KJ702787.1	1,143bp	Sykes, 1832
Columba_janthina_janthina	KM926619.1	17,469bp	Temminck, 1830
Columba_jouyi	NC_031868.1	17,179bp	Stejneger, 1887
Columba_junoniae	EU481986.1	972bp	Hartert, 1916
Columba_guinea	EU481984.1	972bp	Linnaeus, 1758
Columba_palumbus	AY251472.1	768bp	Linnaeus, 1758
Columba_trocaz	EF012590.1	821bp	Heineken, 1829
Columba_bollii	EU481983.1	972bp	Godman, 1872

Columba_leuconota	KJ456239.1	1,019bp	Vigors, 1831
Columba_oenas	EU481988.1	972bp	Linnaeus, 1758
Columba_rupestris	NC_031867.1	17,201bp	Pallas, 1811
Columba_sp_ARA_2012	JX968148.1	1,143bp	Linnaeus, C 1758
Columba_livia	KY378739.1	1,066bp	Gmelin, 1789
Phapitreron_leucotis	AF279712.1	1,045bp	Temminck, 1823
Phapitreron_amethystinus	KT023378.1	1,020bp	Bonaparte, 1855
Phapitreron_cinereiceps	KT023379.1	944bp	Bourns & Worcester, 1894
Claravis_pretiosa	KJ639096.1	986bp	Ferrari-Perez, 1886
Claravis_mondetoura	KJ639093.1	1,067bp	Bonaparte, 1856
Claravis_geoffroyi	KX169277.1	1,069bp	Temminck, 1811
Metriopelia_aymara	KJ639099.1	1,051bp	Prévost, 1840
Metriopelia_melanoptera	KJ639092.1	1,067bp	Molina, 1782
Ocyphaps_lophotes	KU194390.1	943bp	Temminck, 1822
Metriopelia_ceciliae	AF182688.2	1,068bp	Lesson, RP 1845
Metriopelia_morenoi	KJ639083.1	1,067bp	Sharpe, 1902
Uropelia_campestris	EF373288.1	1,044bp	Spix, JB 1825
Columbina_cruziana	AF483318.1	1,052bp	Prevost, 1842
Columbina_picui	KJ639094.1	1,067bp	Temminck, 1813
Columbina_squammata	AF483347.1	1,052bp	Lesson, 1831
Columbina_inca	KJ639103.1	1,038bp	Lesson, 1847
Columbina_passerina	KJ639102.1	1,051bp	Linnaeus, 1758
Columbina_minuta	KJ639100.1	1,067bp	Linnaeus, 1766
Columbina_buckleyi	KJ639079.1	1,027bp	Sclater & Salvin, 1877
Columbina_talpacoti	KJ639101.1	1,067bp	Temminck, 1810
Chalcophaps_indica	MH307428.1	987bp	Linnaeus, 1758
Chalcophaps_stephani	KT023366.1	996bp	Pucheran, 1853
Oena_capensis	AF483353.1	1,044bp	Linnaeus, 1766
Turtur_brehmeri	AY151005.1	1,069bp	Hartlaub, KJG 1865
Turtur_tympanistria	MH307639.1	962bp	Temminck, 1809
Turtur_abyssinicus	KT023399.1	1,020bp	Sharpe, 1902
Turtur_afer	MH307638.1	962bp	Linnaeus, C 1766
Treron_phayrei	KJ456492.1	780bp	Blyth, 1862
Treron_curvirostra	NC_059795.1	17,540bp	Gmelin, JF, 1789
Treron_apicauda	KJ456489.1	406bp	Blyth, 1846
Treron_sieboldii	AY274042.1	1,282bp	Temminck, 1835
Treron_sphenurus	NC_062674.1	18,919bp	Vigors, 1832
Treron_bicinctus	KJ456490.1	918bp	Jerdon, 1840
Treron_phoenicopterus	KJ456493.1	898bp	Latham, 1790
Treron_calvus	MH307626.1	1,030bp	Temminck, 1811
Treron_sanctithomae	MH307629.1	1,030bp	Gmelin, JF, 1789
Treron_griveaudi	MH307627.1	990bp	Benson, 1960
Treron_australis	AF483349.1	1,052bp	Linnaeus, C 1771
Treron_waalia	AF483350.1	1,052bp	Meyer, FAA 1793

Ducula_zoeae	AF483333.1	1,052bp	Desmarest, 1826
Ducula_aenea	AF483331.1	1,052bp	Linnaeus, 1766
Ducula_pacifica	KT023372.1	1,020bp	Gmelin, 1789
Ducula_rufigaster	EF373277.1	1,024bp	Quoy & Gaimard, 1830
Ducula_pinon	AF483332.1	1,052bp	Quoy & Gaimard, 1824
Ducula_bakeri	KT023369.1	1,020bp	Kinnear, 1928
Ducula_bicolor	KT023370.1	1,020bp	Scopoli, 1786
Hemiphaga_chathamensis	GQ912617.1	977bp	Rothschild, 1891
Hemiphaga_novaeseelandiae	NC_013244.1	17,264bp	Gmelin, JF, 1789
Lopholaimus_antarcticus	GQ912619.1	891bp	Shaw, 1793
Gymnophaps_albertisii	EF373280.1	1,044bp	Salvadori, 1874
Ptilinopus_occipitalis	AF483330.1	1,052bp	Gray, 1844
Ptilinopus_magnificus	KT023383.1	1,020bp	Temminck, 1821
Ptilinopus_melanospilus	AF483328.1	1,052bp	Salvadori, 1875
Ptilinopus_superbus	AF483329.1	1,052bp	Temminck, 1809
Ptilinopus_rivoli	KT023395.1	901bp	Prévost, 1843
Ptilinopus_cinctus	KT023380.1	1,020bp	Temminck, 1809
Ptilinopus_porphyreus	KT023388.1	1,020bp	Temminck, 1822
Drepanoptila_holosericea	AF483345.1	1,052bp	Temminck, 1809
Alectroenas_madagascariensis	AF483344.1	1,052bp	Linnaeus, 1766
Alectroenas_sganzini	MH307415.1	986bp	Bonaparte, 1854
Ptilinopus_pulchellus	EF373285.1	1,044bp	Temminck, 1835
Ptilinopus_greyii	KT023381.1	1,020bp	Bonaparte, 1857
Ptilinopus_regina	KT023389.1	1,020bp	Swainson, 1825
Ptilinopus_perousii	KT023386.1	1,020bp	Peale, 1848
